# Echocardiographic assessment of left atrial function for prediction of efficacy of catheter ablation for atrial fibrillation

**DOI:** 10.1097/MD.0000000000027278

**Published:** 2021-09-24

**Authors:** Ewa Pilichowska-Paszkiet, Jakub Baran, Piotr Kułakowski, Beata Zaborska

**Affiliations:** Department of Cardiology, Centre of Postgraduate Medical Education, Grochowski Hospital, Warsaw, Poland.

**Keywords:** ablation, atrial fibrillation, echocardiography, left atrium, strain

## Abstract

Proper selection for catheter ablation (CA) for atrial fibrillation (AF) is still an issue. Echocardiographic assessment of left atrium (LA) is complex and challenging. Speckle tracking echocardiography (STE) with recent standardized LA deformation analysis allows for the quantitative assessment of various LA function parameters. We aimed to assess the value of detailed evaluations of LA function using STE in patients with non-valvular AF without structural heart disease to predict the outcomes after CA for AF. Secondary aim was to analyze the prediction of CA efficacy in patients with normal LA dimension in baseline echocardiography.

We studied with transthoracic and transesophageal echocardiography 82 patients (58% males, mean age 57.3 ± 9.5 years) with non-valvular paroxysmal AF without structural heart disease scheduled for CA. Peak longitudinal LA strain (LAS) and strain rate (LASR) during the reservoir (r), conduit (cd) and contraction (ct) phases were measured by STE before the procedure. Patients were followed for 1 year using serial 4 to 7 day Holter ECG monitoring.

Complete freedom from any AF recurrence was achieved in 44 (54%) patients. All patients had normal left ventricular systolic and diastolic function and 53 (65%) of them had not enlarged LA. In the multivariable logistic regression analysis, global left atrial reservoir strain (LASr) was identified as an independent predictor of CA efficacy (OR [95% CI]: 1.35 [1.17–1.55], *P* < .0001). The opportunity of CA success was 135 fold higher for each 1% increase in global LASr.

The receiver operating characteristic (ROC) analysis identified global LASr and left atrial conduit strain (LAScd) as the most powerful parameters for predicting of CA outcome with an area under the curve of 0.896 and 0.860, respectively, in the whole study group, and 0.922 and 0.938, respectively, in patients with not enlarged LA.

In patients with paroxysmal AF and normal standard echocardiographic assessment, parameters reflecting LA compliance - reservoir and conduit strain, are independent and strong predictors of CA outcome.

## 1 Introduction

Catheter ablation (CA) for atrial fibrillation (AF) is an established therapeutic option; however, post ablation AF recurrence is one of the most important and frequent adverse outcomes, which occurs in 30% to 50% of cases.^[1,2]^ Because results of ablation which has been routinely performed for more than 20 years, are still suboptimal, the identification of subjects who would benefit the most from the procedure is still an issue.^[3]^ Multiple factors including traditional cardiac risk factors, left ventricular (LV) dysfunction, left atrial (LA) enlargement and increased fibrosis have been shown as possible predictors of CA efficacy.^[1,3–7]^ However in patients with not enlarged LA dimensions and normal diastolic as well as LV systolic function, the prediction of arrhythmia recurrence remains a challenge.

Accurate assessment of LV systolic and diastolic function is mandatory part of every echocardiographic examination, while for LA is limited to measuring the size and basic parameters of systolic function. We hypothesized that a detailed, advanced assessment of LA function is crucial for the proper selection of candidates for CA. LA function is complex and therefore requires a multi-parametric assessment. Speckle tracking echocardiography (STE) provides an opportunity to measure various LA function parameters throughout the reservoir, conduit and contractile phases.^[8]^ LA strain has been shown to have predictive value in assessing CA efficacy,^[9,10]^ however small sample sizes of the studies as well as heterogeneity of the studied populations and echocardiographic methods suggest the need for further studies. Recently, the standardization of LA deformation using STE was developed.^[11]^

The aim of our study was to assess the value of detailed assessment of LA function using STE in patients with non-valvular AF without structural heart disease, especially in those with not enlarged LA, in predicting the outcome after CA for AF.

## 2 Methods

### 2.1 Study population

We prospectively screened 208 consecutive patients with AF admitted to our institution between July 2011 and January 2014 for CA. The inclusion criteria were: non valvular AF without structural heart disease and first-time CA. The exclusion criteria were severe valvular heart disease according to European Society of Cardiology guidelines,^[12]^ a LV ejection fraction <40% and poor-quality two-dimension echo images precluding visualization of the LA wall. One hundred 6 patients were not included to the study due to prior CA (47), no possibility to continue follow-up (34), uninterpretable echo images (9), left ventricular ejection fraction <40% (9), congenital heart disease (2), severe mitral regurgitation (2) and LA appendage (LAA) occluder (1). Out of the 102 remaining patients, 18 had AF during index echocardiographic examination and were 2 lost to follow-up which left 82 patients available for analysis. All patients underwent transthoracic and transesophageal echocardiography (transthoracic echocardiography and transesophageal echocardiography [TEE], respectively) within 24 to 48 hours before CA. The study was approved by the local ethics committee (approval number 58/pulsed-wave Doppler/2011). All patients gave written informed consent to participate in the study.

### 2.2 Echocardiography

Transthoracic echocardiography was performed using Vivid 9 (GE Medical System, Horten, Norway, 2010). The cardiac dimensions and LV parameters were measured in accordance with the current recommendations.^[13]^

Parameters of LA size and function are summarized in Table 1. The LA diameter was measured at end-systole in the parasternal long-axis view. The LA volume (LAV) was calculated from the apical 4-chamber (4C) and 2-chamber (2C) views using biplane area-length method. The LAV index was defined as the LAV divided by the body surface area. Mitral flow velocities (E and A) were assessed by pulsed-wave Doppler. Tissue Doppler imaging was used to measure velocities of the early (e’) and late (a’) diastolic phases at the mitral annular septal and lateral corners. The E/e’ ratio was calculated by dividing E by the average of the septal and lateral e’ velocities.

**Table 1:** Echocardiographic parameters of left atrial size and function.

	LA function	
LA size	Diastolic/compliance	Systolic
LA diameter (LAd) LA volume (LAV) index	global LA reservoir strain (LASr) global LA conduit strain (LAScd) global LA reservoir strain rate (LASRr) LA stiffness index (LAstf)	global LA contractile strain (LASct) global LA contractile strain rate (LASRct) Mitral A a’ LA appendage velocity (LAAv)

**Figure 1:**
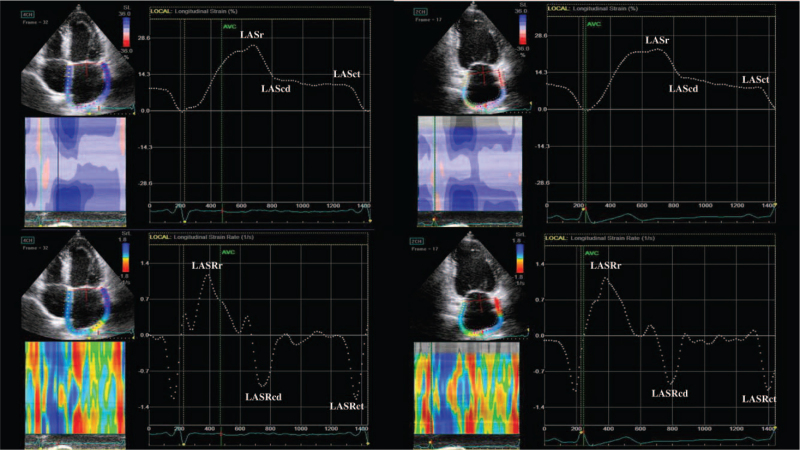
Measurement of peak longitudinal left atrial strain (LAS) and strain rate (LASR) during the reservoir (r), conduit (cd) and contractile (ct) phases obtained from the apical 4 chamber (left panel) and 2 chamber (right panel) views.

Peak longitudinal LA strain (LAS) and strain rate (LASR) during the reservoir (r), conduit (cd) and contraction (ct) phases were measured by STE (Fig. 1). All LAS measurements were analyzed according to the recent consensus document of the European Association of Cardiovascular Imaging/American Society of Echocardiography/Industry Task Force to standardize deformation imaging.^[11]^ The images in the apical 4C and 2C views images were obtained with a frame rate set between 60 and 80 frames per second. Loops of 3 cardiac cycles were stored digitally and analyzed offline with software (EchoPac, GE Healthcare) by an experienced echocardiographer. The LA endocardium was manually traced in the 4C and 2C views to create a region of interest composed of 6 segments in each view. After segmental tracking quality analysis with the possibility of manual adjustments to the region of interest, the software generated strain curves for each atrial segment. The global LAS for each phase were calculated by averaging the values observed in all LA segments. We set the zero strain point at LV end-diastole. The LA stiffness index (LAstf),^[14]^ the ratio of E/e’ to left atrial reservoir strain (LASr), was calculated.

The reproducibility of two-dimension STE was tested in 18 randomly selected patients.^[15]^ LAS was reanalyzed on the digitally stored baseline images at least 3 months later in 18 randomly selected patients by the same observer to evaluate intraobserver variability. To evaluate interobserver variability, a second experienced observer analyzed the same data and was blinded to the other observer's results.

TEE with estimation of the LA appendage velocity (LAAv) was performed according to the standard practice guidelines using Vivid 9 (GE Medical System, Horten, Norway, 2010) with 6T and 6TC multiplane TEE probes.^[16,17]^

### 2.3 Catheter ablation procedure

Patients underwent radiofrequency (RFCA) or cryoballoon (CB) ablation performed according to widely accepted protocols.^[3]^ Allocation to RF or CB ablation was random. Point-by-point pulmonary vein isolation (PVI) using RF energy was performed after double transseptal puncture using irrigated ablation catheters (Thermocool SF or Thermocool Smart Touch ST), a LASSO catheter and the CARTO 3 system (Biosense Webster, USA). CB PVI was performed using a single transseptal puncture. A steerable 15 Fr sheath (FlexCath Advance, Medtronic, Minnesota, USA) was positioned in the left atrium and an inner lumen mapping catheter for PV potential recordings (Achieve, Medtronic, Minnesota, USA) was advanced in each PV ostium. A 28 mm CB (Arctic Front or Arctic Front Advance, Medtronic, Minnesota, USA) was used.

### 2.4 Follow-up

The follow-up lasted 1 year. Patients were seen in the outpatient clinic 3, 6, and 12 months after CA and underwent serial 4 to 7 day Holter ECG monitoring (DMS 300-4A, DM Software, Nevada, USA). The recurrence of arrhythmia was defined as AF or atrial tachycardia (AT) that lasted at least 30 seconds and was documented on standard ECG or during Holter ECG monitoring without taking into account early recurrences corresponding to the blanking period of the first 3 months after the CA.

The definition of CA success was freedom from any symptomatic or asymptomatic AF or AT recorded on ECG or during Holter ECG monitoring without antiarrhythmic (AA) drug therapy or with continued unchanged AA. The remaining patients were classified as having CA failure.

### 2.5 Statistical analysis

Depending on the distribution of continuous variables, the results are presented as means ± SD (normal distribution) or median and quartiles [Q1:25th - Q2:75th percentiles] (non-normal distribution) The distribution of data was examined by the Shapiro–Wilks test as well as by visually assessing histograms and QQ-plots. Dichotomous variables are shown as numbers and percentages. Student *t* test was used for comparison of normally distributed continuous variables, and the Mann–Whitney test for comparison of non-normally distributed continuous variables. Categorical variables were compared using the *X*^2^ test or Fisher Exact test. The *P* value <.05 was taken to indicate a significant difference. The relationship between CA efficacy and potential predictors was assessed using multivariable binary logistic regression analysis (with stepwise method selection and a significance level of 0.05 for entry and 0.05 to stay). All the variables listed in the Table 4 were taken as the potential predictors. The values of LAstf, global LASRr and LAAv were rescaled by multiplying by ten due to better interpretation of odds ratio. The receiver operating characteristic (ROC) comparisons were performed using a contrast matrix to take differences of the areas under the empirical ROC curves (DeLong, DeLong, and Clarke-Pearson). Intra and inter-observer reproducibility was computed by coefficient of variability and intra-class correlation coefficient with 95% confidence interval. Statistical analysis was performed using SAS 9.2 (NC, USA) software.

## 3 Results

### 3.1 Patient characteristics

The study group consisted of 82 patients (58% males, mean age 57.3 ± 9.5 years) with paroxysmal AF who were in sinus rhythm during the analysis. Demographic and clinical parameters of the whole study group as well as comparison between effectively and not effectively treated patients are shown in Table 2. The only significant difference was age which was younger in successfully treated patients.

**Table 2:** Demographic and clinical baseline characteristics of the studied population. Comparison between effectively and not effectively treated patients.

Clinical characteristic	Whole study group N = 82	CA success N = 44 (54%)	CA failure N = 38 (36%)	CA success vs CA failure *P*
Men n (%)	58 (70.7%)	33 (75.0%)	25 (65.8%)	.36
**Age [yr]**	**57.3 ± 9.5**	**54.3 ± 10.6**	**60.7 ± 6.9**	**.002**
BMI [kg/m^2^]	29.8 ± 4.0	29.9 ± 3.8	29.6 ± 4.4	.74
Duration of AF [years]	5.0 [2.7–10.0]	5.8 [3.0–10.0]	4.0 [3.0–10.0]	.74
DM	9 (11%)	3 (6.8%)	6 (15.8%)	.29
CAD	5 (6.1%)	3 (6.7%)	1 (3.7%)	1.00
Arterial hypertension	37 (45.1%)	20 (45.4%)	17 (44.7%)	.95
CHA2DSVASc	1 [0–2]	1 [0–1]	1 [0–2]	.14
Hyperlipidemia	36 (43.9%)	17 (38.6%)	19 (50.0%)	.30
Heart rate [beats/min]	57.0 [53.0–63.0]	56.0 [52.0–63.0]	58.0 [53.0–64.0]	.56
Systolic BP [mm Hg]	132.4 ± 11.8	133.0 ± 10.9	131.8 ± 12.8	.64
Diastolic BP [mm Hg]	84.8 ± 6.9	84.7 ± 6.7	85.0 ± 7.3	.83
Beta-blokers	41 (50%)	20 (45.4%)	21 (55.3%)	.38
AA	13 (15.8%)	6 (13.6%)	7 (18.4%)	.55
ACE-I	39 (47.6%)	19 (43.2%)	20 (52.6%)	.39

AA = antiarrhythmic therapy, ACE-I = angiotensin converting enzyme inhibitor, AF = atrial fibrillation, BMI = body mass index, BP = blood pressure, CA = cathether ablation, CAD = coronary artery disease, DM = diabetes mellitus. Values are expressed as number and (%), mean ± SD, or median and quartiles [Q1:25th–Q2:75th percentiles].

The echocardiographic parameters indicated normal LV systolic and diastolic function. Fifty three (65%) patients had not enlarged (≤34 mL/m^2^) and 19 (23%) - mildly enlarged (35–41 mL/m^2^) LA. The other 5 (6%) patients had moderately (42–48 mL/m^2^) and 5 (6%) - severely (>48 mL/m^2^) enlarged LA. Table 3 shows echocardiographic parameters of the study group.

**Table 3:** Echocardiographic baseline characteristics of the studied population. Comparison between effectively and not effectively treated patients.

Parameter	Whole study group N = 82	CA success N = 44 (54%)	CA failure N = 38 (36%)	CA success vs CA failure *P*
LVEF [%]	66.2 ± 6.4	66.1 ± 7.0	66.3 ± 5.8	.90
IVSDd [mm]	11.0 [10.0–13.0]	11.0 [10.0–12.5]	11.0 [10.0–13.0]	.56
LAd [cm]	38.8 ± 4.2	38.8 ± 4.1	38.9 ± 4.3	.87
LAV index [ml/m2]	29.0 [24.0–38.0]	28.0 [23.0–33.5]	33.0 [25.0–42.0]	.036
Mitral E [cm/s]	69.4 ± 17.2	66.0 ± 19.0	73.3 ± 14.8	.06
Mitral A [cm/s]	55.5 [43.0–65.0]	55.0 [46.0–69.5]	58.0 [40.0–63.0]	.38
e’ [cm/s]	9.0 [8.5–10.0]	9.8 [8.5–10.5]	9.0 [8.5–9.5]	.06
a’ [cm/s]	8.2 ± 2.1	8.8 ± 2.1	7.6 ± 1.9	.007
E/e’	7.9 ± 2.7	7.2 ± 2.5	8.6 ± 2.6	.014
Global LASr [%]	27.1 ± 8.4	32.0 ± 6.1	21.1 ± 6.9	<.001
LAstf	0.28 [0.19–0.40]	0.20 [0.16 – 0.31]	0.37 [0.23–0.64]	<.001
Global LAScd [%]	−15.03 ± 5.47	−17.88 ± 4.75	−11.55 ± 4.16	<.001
Global LASct [%]	−12.10 ± 5.04	−14.31 ± 4.45	−9.38 ± 4.41	<.001
Global LASRr [s^−1^]	1.15 ± 0.24	1.23 ± 0.22	1.04 ± 0.22	<.001
Global LASRct [s^−1^]	−1.41 ± 0.48	−1.57 ± 0.45	−1.20 ± 0.43	<.001
LAAv [m/s]	0.65 ± 0.24	0.71 ± 0.25	0.58 ± 0.21	.011

CA = catheter ablation, IVSDd = interventricular septum diastolic diameter, LAAv = left atrial appendage velocity, LAd = left atrial diameter, LAScd = left atrial conduit strain, LASct = left atrial contractile strain, LASr = left atrial reservoir strain, LASRct = left atrial contractile strain rate, LASRr = left atrial reservoir strain rate, LAstf = left atrial stiffness index, LAV = left atrial volume, LVEF = left ventricular ejection fraction. Values are expressed as the mean ± SD or median and quartiles [Q1:25th–Q2:75th percentiles].

### 3.2 Procedural data

RFCA was performed in 48 (58.5%) patients, and CB was performed in 34 (41.5%) patients. There were no differences in the baseline demographic, clinical and echocardiographic parameters between the RFCA and CB subgroups except for LAVindex (34.0 ± 10.9 vs 27.5 ± 6.5 mL/m^2^, *P* = .001) and left atrial conduit strain (LAScd) (−14.1 ± 5.2 vs −16.7 ± 5.6%, *P* = .043). Complete PVI was achieved in all patients (all PV isolated), and there were no major complications other than local hematoma.

### 3.3 Follow-up results

The CA success was achieved in 44 (54%) patients: no AF/AT off AA was achieved in 37 patients and on continued unchanged AA - in 7 patients. The remaining 38 (46%) patients were classified as having ablation failure. There were no significant differences in the ablation outcomes between patients treated with RFCA and those who underwent CB.

### 3.4 Echocardiographic parameters related to ablation outcome

Table 3 shows a comparison of the echocardiographic parameters between effectively and not effectively treated patients. Patients with successful CA had significantly lower LAV index and significantly better LA strain parameters than those who failed procedure.

In the multivariable logistic regression analysis, the global LASr was identified as an independent predictor of CA success (odds ratio [95% CI]: 1.35 [1.17–1.55], *P* < .001). The opportunity of CA success was 135 fold higher for each 1% increase in global LASr. Table 4 shows results of univariable and multivariable analysis. A global LASr >28% had a high positive predictive value 85.0 (73.9–96.1) with an acceptable negative predictive value 77.5 (64.6–90.4) in identification of patients with successful CA.

**Table 4:** Results of the univariable and multivarible analysis.

	Univariable analysis			Multivariable analysis		
Parameter	OR [95% CI]	AUC	*P*	OR [95% CI]	AUC	*P*
Age [↑ 1 year]	0.917 [0.863–0.975]	0.68 [0.56–0.80]	.005			
LAVindex [↑ 1 ml/m^2^]	0.944 [0.899–0.992]	0.63 [0.51–0.76]	.022			
a’ [↑ 1 cm/s]	1.367 [1.073–1.743]	0.66 [0.54–0.78]	.011			
E/e’ [↑ 1]	0.806 [0.674–0.963]	0.65 [0.53–0.77]	.017			
Global LASr [↑ 1%]	1.323 [1.169–1.498]	0.89 [0.81–0.96]	<.001	1.350 [1.170–1.551]	0.896 [0.822–0.970]	<.001
LAstf [↑ 0.1]	0.461 [0.308–0.691]	0.81 [0.71–0.90]	<.001			
Global LAScd [↑ 1%]	1.420 [1.210–1.666]	0.85 [0.76–0.94]	<.001			
Global LASct [↑ 1%]	1.284 [1.131–1.458]	0.79 [0.69–0.89]	.001			
Global LASRr [↑ 0.10 s^−1^]	1.540 [1.182–2.006]	0.73 [0.61–0.85]	.001			
Global LASRct [↑ 1%]	0.135 [0.039–0.466]	0.73 [0.61–0.84]	.001			
LAAv [↑ 0.10 m/s]	1.298 [1.053–1.600]	0.66 [0.54–0.78]	.014			

AUC = area under the curve, LAAv = left atrial appendage velocity, LAScd = left atrial conduit strain, LASct = left atrial contractile strain, LASr = left atrial reservoir strain, LASRct = left atrial contractile strain rate, LASRr = left atrial reservoir strain rate, LAstf = left atrial stiffness index, LAV = left atrial volume, OR = odds ratio.

**Figure 2:**
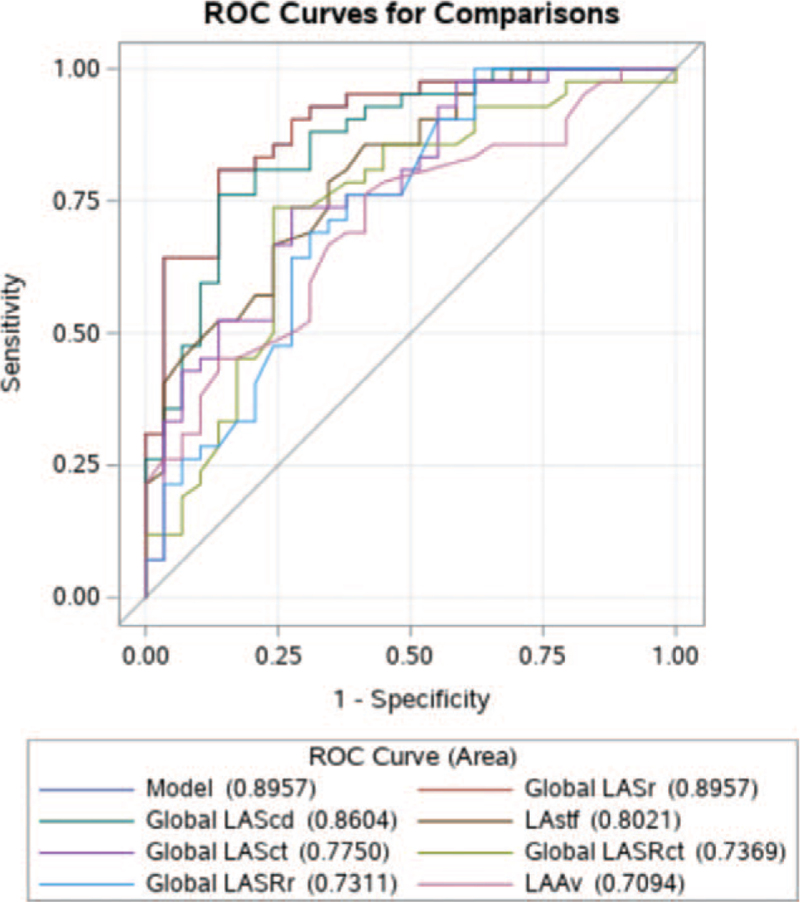
ROC curves for predicting the outcome of catheter ablation for atrial fibrillation.

The ROC curves identified LASr and LAScd as powerful parameters for predicting the CA outcome, with an area under the curve of 0.896 and 0.860, respectively (Figure 2). The ROC comparison analysis showed that LASr was the best parameter and LAScd was not significantly inferior to LASr (*P* = .38) for predicting CA outcome. The other LA function parameters: LAstf, left atrial contractile strain, LASRct, LASRRr, and LAAv, were significantly poorer predictors of CA outcome than LASr (Table 5).

**Table 5:** The ROC comparison analysis in prediction of outcome of catheter ablation.

Contrast	Estimate	Standard error	95%Wald confidence Limits		*P*
Global LAScd - Global LASr	−0.035	0.040	−0.113	0.043	.38
LAstf - Global LASr	−0.094	0.035	−0.162	−0.025	.007
Global LASct - Global LASr	−0.121	0.042	−0.203	−0.038	.004
Global LASRct - Global LASr	−0.159	0.055	−0.266	−0.051	.004
Global LASRr - Global LASr	−0.165	0.055	−0.273	−0.056	.003
LAAv - Global LASr	−0.186	0.060	−0.304	−0.069	.002

LAAv = left atrial appendage velocity, LAScd = left atrial conduit strain, LASct = left atrial contractile strain, LASr = left atrial reservoir strain, LASRct = left atrial contractile strain rate, LASRr = left atrial reservoir strain rate, LAstf = left atrial stiffness index.

### 3.5 Prediction the catheter ablation success in the subgroup with normal left atrial appendage dimension

**Figure 3:**
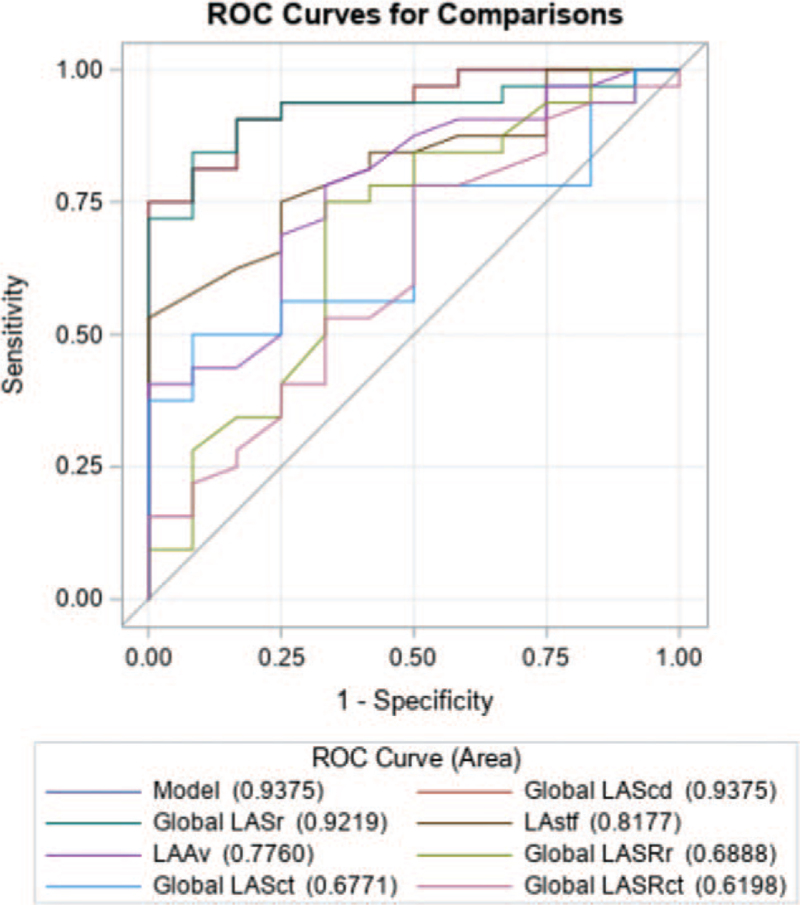
ROC curves for predicting the outcome of catheter ablation for atrial fibrillation in patients with normal left atrial dimension.

Out of the whole study group with the average LAVindex of 31.3 ± 10.0 mL/m^2^, 29 (35%) patients had enlarged LA. We repeated all calculations after excluding these patients. In the multivariable logistic regression analysis, the global LAScd was identified as an independent predictor of CA success (odds ratio [95% CI]: 1.93 [1.28–2.92], *P* = .002). Again, the ROC analysis identified global LAScd and LASr as powerful parameters for predicting CA outcome with an area under the curve of 0.938 and 0.922, respectively (Fig. 3).

The ROC comparison analysis showed that LAScd was the best parameter and LASr was not significantly inferior to LAScd (*P* = .62) for predicting CA outcome. The other LA function parameters were significantly poorer predictors of CA outcome than LAScd (Table 6).

**Table 6:** The ROC comparison analysis in prediction of outcome of catheter ablation in subgroup with not enlarged LA.

Contrast	Estimate	Standard error	95% Wald confidence Limits		*P*
Global LASr - Gobal LAScd	−0.0156	0.0312	−0.0768	0.0455	.62
LAstf - Global LAScd	−0.1198	0.0547	−0.2269	−0.0127	.028
Global LASct - Global LAScd	−0.2604	0.0862	−0.4294	−0.0915	.003
Global LASRct -Global LAScd	−0.3177	0.0987	−0.5111	−0.1243	.001
Global LASRr - Global LAScd	−0.2487	0.0940	−0.4330	−0.0644	.008
LAAv - Global LAScd	−0.1615	0.0909	−0.3397	0.0167	.076

LAAv = left atrial appendage velocity, LAScd = left atrial conduit strain, LASct = left atrial contractile strain, LASr = left atrial reservoir strain, LASRct = left atrial contractile strain rate, LASRr = left atrial reservoir strain rate, LAstf = left atrial stiffness index.

### 3.6 Feasibility and reproducibility of left atrial appendage strain measurements

Out of images initially classified as interpretable, the measurement of LAS was not feasible in 3 (3.6%) patients. The LAS had very good intra-observer and inter-observer reproducibility (Table 7).

**Table 7:** Intra- and inter-observer reproducibility.

	Intra-observer		Inter-observer	
	COV (%)	ICC (95% CI)	COV (%)	ICC (95% CI)
LASr 4C	3.1%	0.995 [0.992–0.998]	4.3%	0.993 [0.988–0.996]
LASr 2C	2.5%	0.998 [0.996–0.999]	2.9%	0.997 [0.995–0.999]
LASct 4C	4.6%	0.995 [0.992–0.998]	4.7%	0.995 [0.991–0,997]
LASct 2C	3.4%	0.997 [0.995–0.998]	2.9%	0.998 [0.996–0.999]

CI = confidence interval, COV = coefficient of variability, ICC = intra-class correlation, LASct 2C = left atrial contractile strain in two chamber view, LASct 4C = left atrial contractile strain in four chamber view, LASr 2C = left atrial reservoir strain in two chamber view, LASr 4C = left atrial reservoir strain in four chamber view.

## 4 Discussion

The major finding of our study is that out of many echocardiographic variables that describe LA function, parameters reflecting LA compliance – LASr and LAScd are the strong and independent predictors of the outcome of CA for paroxysmal AF in patients with normal standard echocardiography.

Multiple factors have been shown to be predictors of efficacy of CA. Previous reports demonstrated that LA enlargement was a strong predictor of AF recurrence after CA^[18]^ although the usefulness of this parameter is limited. The LAV can be increased in patients with diastolic dysfunction or bradycardia, in trained athletes but may decrease as a result of therapy with diuretics. In the present study, there was a significant difference in LAVindex between the CA failure and success groups, although the multivariable analysis did not identify LAVindex as an independent predictor of efficacy of CA. The LA size is one of the most frequently used parameters for selecting patients for CA of AF, however, CA may fail in a significant proportion of them as was shown in the our study, what indicates that thorough LA function assessment, not dimension, may be crucial in selecting patients for CA. An interesting finding of our study is the fact that echocardiographic parameters reflecting LA compliance occurred useful also in a subgroup with normal LA dimensions. Thus, the advent of new echocardiographic techniques reflecting LA compliance which is altered by LA fibrosis may improve identification of responders to CA and help making decision as to perform CA.

It has been shown that increased LA fibrosis which can be already present in not enlarged LA and in patients with lonely AF, was significantly associated with AF recurrence post CA.^[4]^ A reduced LAS during the reservoir phase has been shown to correlate with histopathological alterations of the LA wall and the degree of fibrosis estimated by late gadolinium enhancement magnetic resonance imaging.^[4]^ We also previously showed that LASr and LAstf correlated well with the extent of LA fibrosis assessed invasively using electroanatomical mapping and found stronger associations between low atrial potential areas and the parameters characterizing LA compliance (LASr, LAstf) than between the same areas and the parameters characterizing LA systolic function (left atrial contractile strain, LAAv, A, a’).^[15]^

The LA mechanics in predicting the outcome after CA in patients with AF have been analyzed in several studies. Koca et al reported that global LAS and LAV index were independent parameters predicting AF recurrence after cryoablation with the cutoff value of 18.1%, with sensitivity of 92.6% and specificity of 85.7%.^[19]^ However, authors did not take into consideration the complexity of the LA function and out of LA deformation parameters only reservoir strain was analyzed.

Consistent with previous results, our study indicated that LASr has a high prognostic value as a predictor of AF recurrence after CA.^[20]^ Ma et al analyzed in the meta-analysis clinical relevance of LAS to predict recurrence of AF after CA in 8 studies and documented the usefulness of LAS in identifying patients with high risk of AF recurrence after CA. However, out of 8 analyzed studies, 6 examined less homogenous populations compared to our study because both paroxysmal and persistent AF patients were included. During AF, LA function during the reservoir and conduit phases is severely impaired, and systolic function does not exist: hence a reduction in LAS is observed. Reduced LAS in AF occurs mainly due to atrial mechanical function impairment (lack of systole, impairment of diastole) rather than as a reflection of atrial wall properties. We previously reported no significant relationships between low atrial potential areas and LA function parameters in patients examined during AF.^[15]^ Our present study included only patients with sinus rhythm.

Two studies included in the above-mentioned meta-analysis investigated patients with paroxysmal AF, however there are some differences when comparing with our study. Hwang et al demonstrated that lower contractile LAS was strongly associated with AF recurrence after CA^[21]^ however the study group included only 40 patients and follow-up lasted 9 months. Morris et al showed that both LA diastolic and systolic dysfunction could be useful in distinguish patients with high or low risk of recurrence of AF after CA and found LAS 188% to be cut-off value.^[22]^

Although LAS has been widely used in clinical studies, there were inconsistencies and pitfalls with these assessments. Recently, the standardization of LA deformation using STE has been developed^[11]^ and shed new light on the results of previous studies. The present study was performed in accordance with the consensus document established by the European Association of Cardiovascular Imaging/American Society of Echocardiography/Industry Task Force.

Although the patients included in our study had AF ablation performed from 7 to 10 years ago, we believe that our results remain valid also nowadays. It is true that efficacy and safety of AF ablation improved over the last decade^[23]^ however, it was mainly due to increasing experience and more effective point-by-point RF application delivery. The type of RF catheters, Electroanatomical mapping software, PVI strategy as well as cryoballoon type and procedural issues were in our study essentially the same as today so there are good reasons to believe that our results are still valid in contemporary ablation field.

Our study showed that detailed echocardiographic assessment of LA function prior to CA for AF may play a major role in selecting patients for this procedure. We showed high predictive value of LASr and LAScd – parameters reflecting LA compliance and diastolic function. All the more, structural fibrotic changes in LA can be present at the very early stage of the disease,^[24]^ traditional echocardiographic images can be normal and modern echocardiography can be effectively used to identify responders to CA of AF. Tests for identification of the best candidates for AF ablation are still sparse. Echocardiographic methods are very promising because the technique is widely accessible, non-invasive and reproducible. Thus, thanks to the recent standardization of the LA deformation assessment, further research should be undertaken for development of reference ranges and defining cut-off values for LA dysfunction in larger populations. As a result, LA function parameters derived from STE could become a standard element of LA assessment. That could be useful not only in patients undergoing AF ablation but wider in the diagnosis of atrial cardiomyopathy.

## 5 Limitations

First, the study group was relatively small, and duration of follow-up was relatively short. However, the follow-up period was completed in all but 2 patients, and the number of patients was sufficient to perform meaningful statistical analysis.

Second, the left atrial conduit strain rate was excluded from the analysis due to difficulties in obtaining high-quality LASR curve in all patients. However, the 2 other LASR parameters - LASRr and LASRct, were significantly inferior to LASr and LAScd in predicting CA success.

Third, although we performed 3 serial 4 to 7 days Holter ECG recordings during a one-year follow-up and patients were frequently seen in the outpatient clinic, we might have missed silent episodes of AF because no long-term continuous ECG recordings were used.

Finally, we used 2 techniques for CA of AF – RFCA and CB, which might have influenced the results. However, the outcomes of CA of AF were similar in both groups and there were only a few minor differences in the baseline echocardiographic parameters between the 2 groups.

## 6 Conclusions

LAS analysis seems to be very important in selecting candidates for CA in patients with AF without abnormalities in standard echocardiographic assessments. Out of many LA function parameters, these reflecting LA compliance - LASr and LAScd, are strong and independent predictors of CA outcome.

## Acknowledgments

We thank Ilona Kowalik for his guidance in the statistical analysis.

## Author contributions

**Conceptualization:** Ewa Pilichowska-Paszkiet, Jakub Baran, Piotr Kulakowski, Beata Zaborska.

**Data curation:** Ewa Pilichowska-Paszkiet, Jakub Baran, Beata Zaborska.

**Formal analysis:** Ewa Pilichowska-Paszkiet, Piotr Kulakowski, Beata Zaborska.

**Funding acquisition:** Ewa Pilichowska-Paszkiet.

**Investigation:** Ewa Pilichowska-Paszkiet, Jakub Baran, Beata Zaborska.

**Methodology:** Ewa Pilichowska-Paszkiet, Jakub Baran, Piotr Kulakowski, Beata Zaborska.

**Project administration:** Ewa Pilichowska-Paszkiet, Piotr Kulakowski, Beata Zaborska.

**Software:** Ewa Pilichowska-Paszkiet.

**Supervision:** Piotr Kulakowski, Beata Zaborska.

**Validation:** Ewa Pilichowska-Paszkiet.

**Writing – original draft:** Ewa Pilichowska-Paszkiet, Piotr Kulakowski, Beata Zaborska.

**Writing – review & editing:** Ewa Pilichowska-Paszkiet, Jakub Baran, Piotr Kulakowski, Beata Zaborska.
